# Characterization of the interaction between HMGB1 and H3—a possible means of positioning HMGB1 in chromatin

**DOI:** 10.1093/nar/gkt950

**Published:** 2013-10-23

**Authors:** Matthew Watson, Katherine Stott, Harry Fischl, Laura Cato, Jean O. Thomas

**Affiliations:** Department of Biochemistry, University of Cambridge, 80 Tennis Court Road, Cambridge CB2 1GA, UK

## Abstract

High mobility group protein B1 (HMGB1) binds to the internucleosomal linker DNA in chromatin and abuts the nucleosome. Bending and untwisting of the linker DNA results in transmission of strain to the nucleosome core, disrupting histone/DNA contacts. An interaction between H3 and HMGB1 has been reported. Here we confirm and characterize the interaction of HMGB1 with H3, which lies close to the DNA entry/exit points around the nucleosome dyad, and may be responsible for positioning of HMGB1 on the linker DNA. We show that the interaction is between the N-terminal unstructured tail of H3 and the C-terminal unstructured acidic tail of HMGB1, which are presumably displaced from DNA and the HMG boxes, respectively, in the HMGB1-nucleosome complex. We have characterized the interaction by nuclear magnetic resonance spectroscopy and show that it is extensive for both peptides, and appears not to result in the acquisition of significant secondary structure by either partner.

## INTRODUCTION

High mobility group protein B1 (HMGB1) is a relatively abundant and versatile nuclear protein that binds to chromatin in a highly dynamic manner (1). It modulates chromatin structure through interactions with DNA and chromatin proteins, interacts with components of the basal transcription machinery and enhances the binding of several transcription factors to their cognate DNA [reviewed in (2–6)]. It relaxes chromatin structure and enhances transcription from chromatinized templates *in vivo* (7).

HMGB1 binds to the nucleosomal linker DNA in the vicinity of the dyad and may be able to displace/replace the linker histone (1,8–12). In addition, HMGB1 has been proposed to ‘prime' the nucleosome core, by stabilizing a bulge/bend in the DNA at the entry/exit point (13), providing a preferential binding site for remodelling complexes (14) and altering the accessibility of nearby transcription factor binding sites (15). ‘Priming' is likely to involve the breaking of several core histone-DNA contacts, through distortion of the DNA on the nucleosome surface and displacement of histone tails on binding of HMGB1 to the linker DNA. The exposed positive charges could in principle be neutralized by the acidic C-terminal tail of HMGB1 (13). The acidic tail is necessary for efficient stimulation of chromatin remodelling (14) and transcription (16,17) by HMGB1. Further, it has been proposed that an interaction between the acidic tail of HMGB1 and the N-terminal tail of H3 might act to position the protein correctly on the nucleosomal linker DNA (17,18).

**Figure 1.**
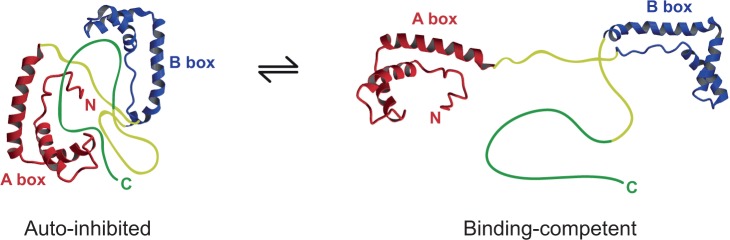
Dynamic association of domains in HMGB1. Schematic indicating the dynamic equilibrium between closed (auto-inhibited) and open (binding-competent) conformations of full-length HMGB1 (only the fully closed and open structures are shown for simplicity). HMG-box DNA-binding domains in red and blue, basic N-terminal, inter-box- and C-terminal extensions in yellow and acidic part of the C-terminal tail in green. [Adapted from (20)].

Our recent work has demonstrated that the acidic tail organizes the HMG boxes and linkers into an ‘auto-inhibited’ complex in which the DNA-binding faces of the boxes are occluded (Figure 1; 19,20). Binding of other partners competes with the intramolecular interactions to promote more of the open, ‘binding-competent’, forms of the protein, thereby liberating domains that were previously sequestered (11,21). This could also be the case for the interaction between HMGB1 and H3 in a chromatin context. Here we confirm that HMGB1 interacts with H3 in chromatin and specifically with the N-terminal tail; we have used linker-histone-depleted chromatin, since HMGB1 and H1 binding may be mutually exclusive (11). We present a detailed characterization of the interaction between HMGB1 and the N-terminal tail peptide of H3, facilitated by the use of a series of HMGB1 tail-truncation mutants (19).

In the absence of DNA or chromatin, the results of chemical cross-linking are in accord with previous observations that the 5–10 C-terminal residues of HMGB1 are necessary for interaction with H3 (17,18). However, in chromatin, we show that the interaction involves the entire length of the HMGB1 tail, presumably because binding of the HMG boxes to DNA outcompetes the intramolecular interactions between the acidic tail and the boxes (Figure 1; 19,20) and also displaces the H3 tail from the linker DNA, allowing the two intrinsically disordered tails to interact. We use nuclear magnetic resonance (NMR) chemical-shift perturbation mapping and circular dichroism (CD) to characterize the interaction between the two tail peptides, which reveals an extensive interface between them and a lack of defined order in the complex.

## MATERIALS AND METHODS

### Protein expression and purification

pGEX2TL-H3(1–40) was created by inserting a stop codon (TAA) into plasmid pGEX2TL-H3 (22) at position 41 in the H3 amino acid sequence using QuickChange site-directed mutagenesis (Stratagene). Glutathione-S-transferase-tagged H3(1–40) was expressed in *Escherichia coli* BL21(DE3) cells grown in LB medium supplemented with 50 µg/ml carbenicillin. Cells were grown at 37°C to an OD_600_ of 0.9. Expression was induced by addition of isopropyl-β-d-thiogalactopyranoside (IPTG) to a final concentration of 1 mM and the cells were grown for a further 5 h at 37°C or overnight at 16°C. Proteins in the cell lysate were bound to glutathione superflow resin (Generon), and the untagged peptide was released by cleavage with thrombin (GE Healthcare). The peptide was further purified on a 1-ml Resource S column using a linear salt gradient (0–1 M) over 20 column volumes.

HMGB1, the tail-truncation proteins and the HMGB1 acidic tail peptide were expressed and purified as described (19). Unlabelled proteins were expressed in M9S medium (23), with the exception of Δ30, which was expressed in 2× yeast tryptone medium. His-tag was cleaved from the acidic tail peptide using thrombin (GE Healthcare), leaving the residual sequence GSM at the N-terminus.

^15^N-labelled proteins were expressed and purified in the same way, but cells were grown in 3-(N-morpholino)propanesulphonic acid (MOPS) minimal medium supplemented with 6 mM ammonium chloride as the sole nitrogen source.

The pure proteins and peptides were concentrated and exchanged into 10 mM sodium phosphate, pH 7.0, 1 mM EDTA, 1 mM dithiothreitol (DTT) [omitted for sulfo-SBED labelling (see below)] using a Vivaspin2 concentrator with a 3 or 10 kDa molecular mass cutoff (Sartorius). Protein concentrations were determined by amino-acid analysis (performed by Mr Peter Sharratt, Protein and Nucleic Acid Chemistry Facility, Department of Biochemistry, University of Cambridge).

### Chromatin isolation

Medium-length chicken erythrocyte chromatin (10–15 nucleosomes) was prepared by micrococcal nuclease digestion and sucrose gradient fractionation, and stripped of linker histones H1 and H5 using 0.6 M NaCl (24). To remove the core histone tails, the chromatin was treated with trypsin (Sigma) at 1:100 (w/w) (enzyme:histone) for 30 min at room temperature. The reaction was stopped with soybean trypsin inhibitor (Type I-S; Sigma) at a 10:1 (w/w) excess over trypsin, and phenylmethylsulphonyl fluoride to a final concentration of 1 mM. Chromatin was dialysed into 10 mM Tris–HCl, pH 7.5, 1 mM EDTA (for sucrose gradients) or 10 mM sodium phosphate, pH 7.0, 1 mM EDTA (for cross-linking and label transfer). Removal of tails was checked by sodium dodecyl sulphate (SDS)/18%-polyacrylamide gel electrophoresis (PAGE) and Coomassie Brilliant Blue staining (25).

### Sucrose gradient sedimentation of HMGB1 and intact or trypsinized chromatin

One A_260_ unit of medium-length linker-histone-depleted chromatin was incubated with HMGB1 on ice for 30 min (in 10 mM Tris-HCl, pH 7.5, 1 mM EDTA). One-tenth of the sample was removed as ‘input’ and the remainder was centrifuged at 4°C through 17 ml linear 5–30% (w/v) sucrose gradients in the same buffer in a Beckman SW40 rotor for 15 h at 20 000 rpm. The gradients were fractionated and monitored at 280 nm. Fractions across the gradient were treated with 25% (final) trichloroacetic acid and proteins in the precipitates were analysed by SDS/18%-PAGE (24).

### Sulfo-SBED label transfer from HMGB1 (and truncations) to chromatin

HMGB1 and its tail-truncated derivatives were labelled using the sulfo-N-hydroxysuccinimidyl-2-(6-[biotinamido]-2-(p-azidobenzamido)-hexanoamido)ethyl-1,3′-dithiopropionate (Sulfo-SBED) Biotin Label Transfer Kit (Pierce) according to the manufacturer’s instructions. A 5-fold molar excess of Sulfo-SBED over HMG protein was used (resulting in labelling of a fraction of the 43 lysines in the protein). Proteins (20 µM) were incubated with linker-histone-depleted chromatin (in 10 mM sodium phosphate, pH 7.0, 1 mM EDTA) at a molar ratio of one HMG per nucleosome in a 10-µl reaction volume for 20 min. Samples were ultraviolet (UV)-irradiated at 312 nm for 15 min on ice at a distance of 5 cm using a transilluminator (Fotodyne). The disulphide bond in the label was then cleaved by treatment with 50 mM tris(2-carboxyethyl)phosphine at 65°C for 10 min. Proteins were resolved by SDS/18%-PAGE and transferred to nitrocellulose. The biotin label was detected by probing with both streptavidin (HRP-conjugated; Pierce) and HRP-conjugated anti-streptavidin antibody (Abcam; ab7239). The identity of the H3 band was confirmed by stripping and re-probing with anti-H3 antibody (Abcam; ab1791) and HRP-conjugated secondary antibody. In both cases, bound antibody was detected using enhanced chemiluminescence substrate (Pierce).

### EDC cross-linking of HMGB1 (and truncations) to chromatin

HMGB1 and its tail-truncated derivatives (10 µM) and linker-histone-depleted chromatin in 10 mM sodium phosphate, pH 7.0, 1 mM EDTA were incubated at a molar ratio of one HMG molecule per nucleosome in a 20-µl reaction volume for 20 min and then treated with 2 mM 1-ethyl-3[3-dimethylaminopropyl]carbodiimide (EDC) for 2 h at 23°C. The reaction was stopped by heating at 100°C in SDS sample buffer, and proteins were resolved by SDS/18%-PAGE and either stained with Coomassie Brilliant Blue (25), or transferred to nitrocellulose and probed with anti-H3 (Abcam; ab1791) or anti-HMGB1 (Abcam; ab12029) antibodies; detection was as above.

### EDC cross-linking of HMGB1 (and truncations) to H3(1–40)

HMGB1 and its tail-truncated derivatives and H3(1–40) each at 10 μM were incubated for 20 min at 23°C in the absence or presence of 25 ng of sonicated calf thymus DNA. Samples were treated with EDC as above, and proteins resolved by SDS/20%-PAGE and visualized with Coomassie Brilliant Blue (25).

### CD spectroscopy

Proteins were dialysed extensively against 10 mM sodium phosphate, pH 7.0, and accurate protein concentrations determined by amino acid analysis. Spectra were recorded for H3(1–40) and the acidic tail peptide (each 20 μM), and a 1:1 mixture of the two (final concentration 10 μM of each). Measurements were made using an AVIV 410 spectropolarimeter at 25°C in a 1-mm path length quartz cuvette. Each curve is the average of five scans recorded with a wavelength step of 1 nm and an averaging time of 5 s after subtraction of a buffer blank.

### NMR spectroscopy

NMR measurements were made on ^15^N-labelled proteins (0.2–0.5 mM) in 10% D_2_O, 10 mM sodium phosphate, pH 7.0, 1 mM EDTA, 1 mM DTT. Experiments were recorded at 25°C (^15^N-HMGB1) or 0°C (^15^N-H3) on a Bruker DRX600 spectrometer. Data were processed using the AZARA suite of programs (v. 2.8, © 1993–2013; Wayne Boucher and Department of Biochemistry, University of Cambridge, unpublished). Assignments were made in CcpNmr Analysis v. 2.1 (26) with reference to previous data for H3(1–59) (27) and HMGB1 (19). Additional 3D NOESY-^15^N-HSQC experiments (28) were used where necessary to confirm assignments and resolve ambiguities. For chemical-shift perturbation experiments, unlabelled proteins, in an identical buffer, were titrated into the appropriate ^15^N-labelled sample to the molar ratio stated. Chemical-shift differences were calculated using Δδ = [(Δδ^H^)^2 ^+ (0.15 × Δδ^N^)^2^]^1/2^ (29). {^1^H}^15^N heteronuclear nuclear Overhauser effect (NOE) values (*I*_sat_/*I*_unsat_) were obtained at 500 MHz using either 4 s of ^1^H saturation by a 120° pulse train or a 4 s delay before the first ^15^N pulse (30); errors were estimated from the standard deviation of the noise.

## RESULTS

### Interaction of HMGB1 with histones in chromatin

Several studies have investigated the interaction of HMGB1 with the core histones, both free in solution (17,18,31,32) and in nucleosomes (17,33,34). In addition, a series of tail-truncated mutants has been used to examine the interaction of HMGB1 with free core histones (17,18). However, no clear overall consensus has emerged as to which core histones and which domains of HMGB1 are involved, possibly due to differences in methodology in the different studies*.*

**Figure 2.**
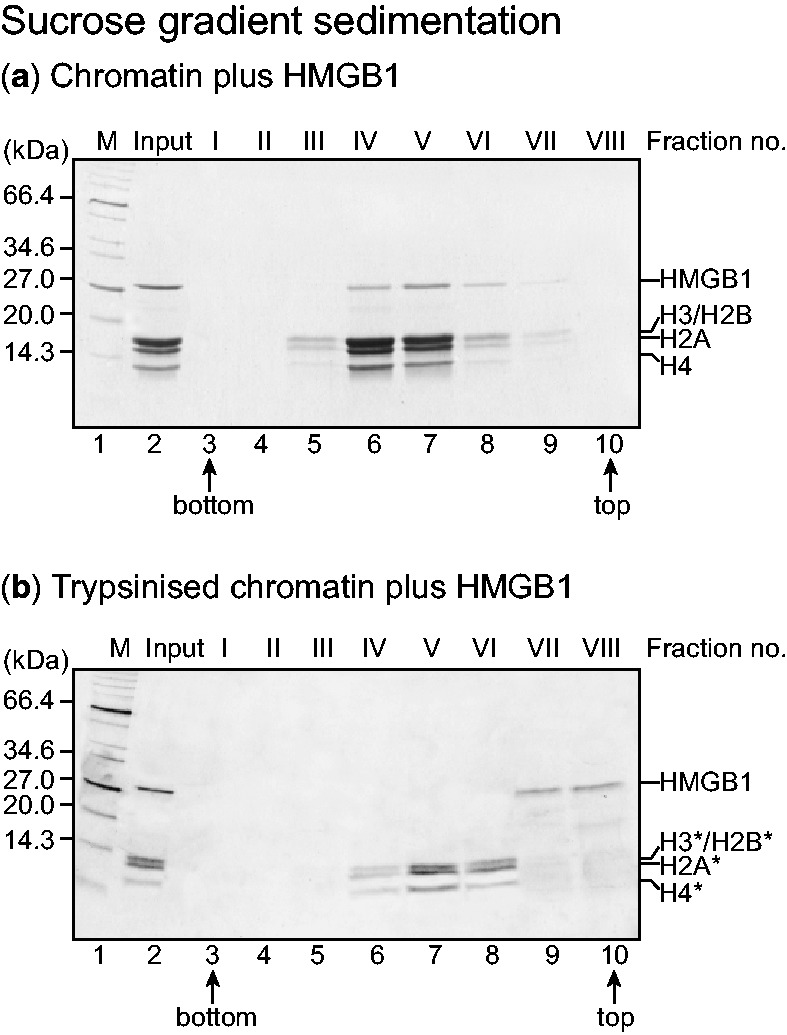
Interaction of HMGB1 with histone tails in chromatin. Sucrose gradient sedimentation of (linker-histone-depleted) chromatin pre-incubated with HMGB1, analysed in SDS/polyacrylamide gels. (**a**) Medium-length chromatin; (**b**) medium-length trypsinized chromatin from which the four core histone tails have been removed (denoted by asterisk). Lanes 3–10 correspond to fraction numbers I–VIII (bottom to top of gradient). Lane 1, molecular mass marker; lane 2, input chromatin.

In a preliminary experiment to determine whether, in a chromatin context, the N-terminal tails of the core histones affect HMGB1 binding, linker-histone-depleted chromatin, and chromatin from which the four core histone N-terminal tails had been removed by digestion with trypsin, were incubated with HMGB1 and sedimented through a sucrose gradient. Only the chromatin with intact core histone tails binds HMGB1; in the absence of the tails, HMGB1 remains at the top of the gradient (Figure 2). Therefore, one or more of the core histone tails are required for stable binding of HMGB1 to chromatin.

**Figure 3.**
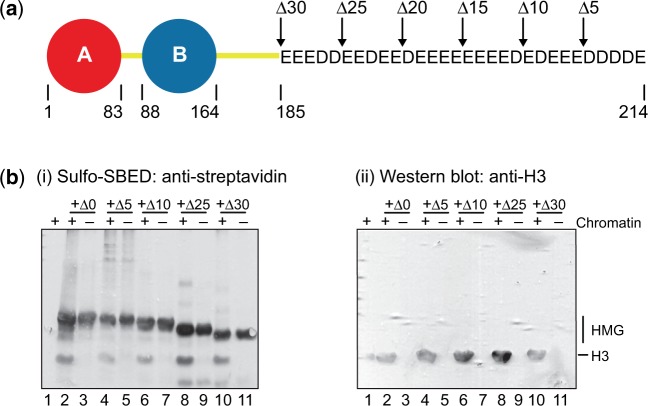
Interaction of HMGB1 and tail-truncated derivatives with chromatin. (**a**) Schematic of HMGB1. The sequence of the C-terminal intrinsically disordered acidic tail is shown, with the positions of the various truncations. (**b**) Label-transfer for HMGB1 and its truncated products modified with the biotin label-transfer reagent Sulfo-SBED, either alone or after incubation with linker-histone-depleted chromatin. After cross-linking, and cleavage of the reagent, proteins were resolved by SDS/PAGE and transferred to nitrocellulose membrane. The biotin label was detected by probing sequentially with streptavidin and anti-streptavidin (i), and the identity of the H3 band was confirmed by stripping and re-probing the same blot with anti-H3 (ii).

To gain insights into which of the core histones interact with (or are in proximity to) HMGB1 in chromatin, we used biotin-label transfer from labelled HMGB1 to its interacting partner(s). To gain additional insights into the role of the acidic tail of HMGB1 we also studied a series of tail-truncated mutants (Figure 3a; 19). HMGB1 and its tail-truncated derivatives were randomly modified at lysine residues in the basic boxes and linkers with Sulfo-SBED, bound to linker-histone-depleted chromatin and the complex exposed to UV light to activate an aryl-azide group in the reagent, which then reacts with HMGB1-interacting partners, thus cross-linking them to HMGB1. Reduction of a disulphide bond in the reagent results in transfer of a biotin label to the interacting partner, which is then detected using streptavidin and an anti-streptavidin antibody. The biotin label was transferred from HMGB1 to H3, but not significantly to any of the other core histones [Figure 3b(i), lane 2]; the identity of the H3 band was confirmed by probing with an antibody specific for H3 [Figure 3b(ii), lane 2]. A significant proportion of the biotin label remained on the HMGB1 protein, presumably due to intramolecular cross-linking, rather than inefficient cleavage of the disulphide (labelled HMGB1 treated with reducing agent without being exposed to UV was not detected; data not shown); this intramolecular label transfer was also observed for UV-irradiated HMGB1 alone [Figure 3b(i) lane 3]. The much lower proportion of label transferred to H3 is not unexpected since many of the modified lysine residues are likely to be distant from the H3/HMG interaction interface.

To investigate the effect of acidic tail length, especially in light of a report that the C-terminal 5–10 residues of the tail were essential for HMGB1 interaction with H3 and correct positioning on the linker DNA (17,18), we tested HMGB1 mutant proteins with progressively shorter acidic tails (HMGB1Δ5, HMGB1Δ10, HMGB1Δ25) or no tail (HMGB1Δ30). In all cases, label transfer to H3 was detected [Figure 3b(i) and (ii) lanes 4, 6, 8 and 10]. In the case of HMGB1Δ25, the additional lower band is unlikely to be H4 because it is present in both the + and − chromatin lanes, and is more likely to be due to a degradation product.

**Figure 4.**
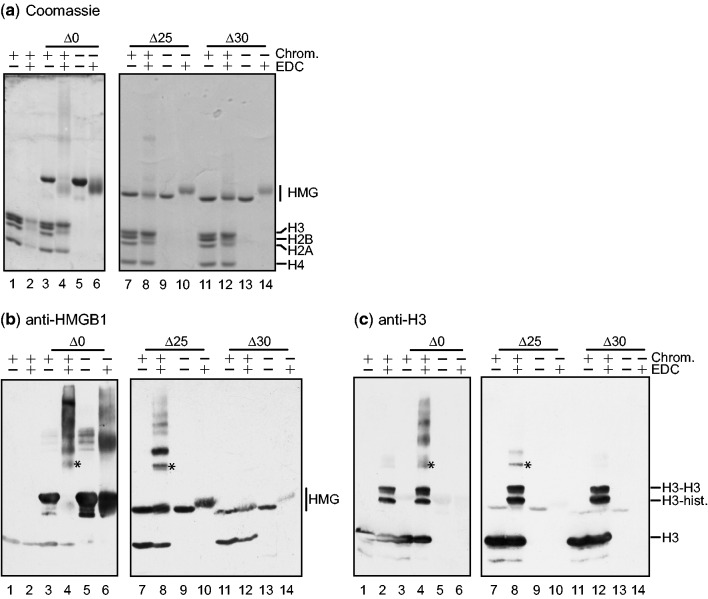
EDC cross-linking of HMGB1 and tail-truncated derivatives to H3 in chromatin. Linker-histone-depleted chromatin alone (lanes 1,2), chromatin with HMG proteins (lanes 3 and 4, 7 and 8, 11 and 12) and HMG proteins alone (lanes 5 and 6, 9 and 10, 13 and 14), untreated or treated with EDC, were resolved by SDS/PAGE and detected by Coomassie staining (**a**). Western blot of identical gels probed with (**b**) anti-HMGB1, (**c**) anti-H3 antibodies, to identify products containing both HMGB1 and H3. Asterisks mark the bands of highest mobility that were detected by both antibodies and probably represent HMG-H3 heterodimers.

To investigate more directly the role of the acidic tail in the interaction with H3, linker-histone-depleted chromatin was incubated with HMGB1 or the tail-truncated proteins and treated with EDC, which cross-links carboxy and amino side-chains. Much of the cross-linking (most apparent as a smear in Figure 4a, lane 4) is likely to be due to histone-histone contacts, which are much more stable than the transient HMG-chromatin interaction. [The increased mobility of full-length HMGB1 (Δ0) after treatment with EDC has been observed previously (11,17,18,35) and is attributed to intramolecular cross-linking (Figure 4a, lanes 4, 6).] To test for cross-linked products containing both H3 and HMG proteins, Western blots were probed with anti-H3 and anti-HMGB1 antibodies. It is clear that several of the lower-mobility cross-linked products indeed contain both. The complexes indicated by asterisks for HMGB1 and HMGB1Δ25 (but not the tailless protein Δ30) (Figure 4b and c) are likely to be HMG-H3 heterodimers; the composition of the higher molecular mass products is unclear. (The two strong bands detected by anti-H3, running slightly slower than HMGB1 and also present in chromatin alone (Figure 4c), are probably H3-H3 and H3-core histone dimers.) Since cross-linked HMG-H3 dimers were still evident for H3 and HMGB1Δ25, which contains only five tail residues (Δ25; Figure 4a lane 8), but not with the tailless protein (Δ30; Figure 4a lane 12), it is clear that even short tails are sufficient for HMG-H3 interaction. The distal 5–10 residues of the 30-residue HMGB1 tail, essential for interaction of H3 and HMGB1 in the absence of chromatin/DNA (17,18), therefore appear to be dispensable for interaction with H3 when HMGB1 is bound to chromatin, presumably because binding of HMG boxes to DNA displaces the acidic tail, leaving this free to interact with other partners (here, H3).

### In the presence of DNA the HMGB1 acidic tail is free to interact with H3(1–40)

**Figure 5.**
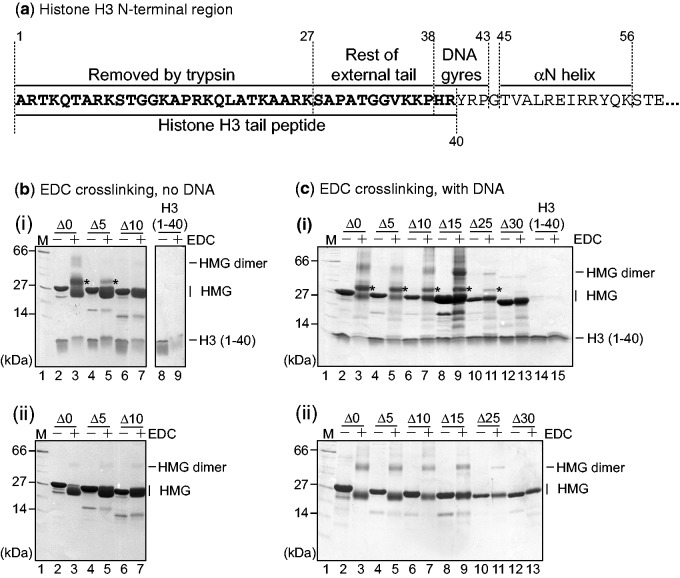
EDC cross-linking of HMGB1 and tail-truncated derivatives to H3(1–40); the effect of DNA. (**a**) Schematic of the H3 N-terminal region, including the region removed by trypsin, the region that passes between the two gyres of DNA and the N-terminal helix. The construct used here, H3(1–40), is indicated below, and roughly corresponds to the region of the tail that extends beyond the two gyres of DNA in the nucleosome. (**b**) (i) EDC cross-linking of HMG proteins and H3(1–40), or H3(1–40) alone; and (ii) the corresponding HMG proteins in the absence of H3. (**c**), as (b) except in the presence of sonicated calf thymus DNA. Lane 1, molecular mass marker. Asterisks indicate H3(1–40)-HMG heterodimers. Samples were resolved by SDS/PAGE and detected by Coomassie Blue staining.

Since the evidence taken together points to a role for the N-terminal tail of H3 in interaction with HMGB1, and since the H3 tail is probably displaced from the linker DNA by the HMG boxes, we studied the interaction of an H3 N-terminal tail peptide (residues 1–40) with HMGB1, as well as with its tail-truncated derivatives. H3(1–40) (Figure 5a) roughly corresponds to the N-terminal intrinsically disordered tail of H3 that is exposed beyond the two DNA gyres in the nucleosome core particle (36) and appears to be sufficient for interaction with full-length HMGB1 (37). In light of the effect of DNA in releasing the acidic tail from the HMG boxes (19,20,38), we studied the interaction in the absence and presence of DNA.

In both cases, EDC cross-linking of full-length HMGB1 with H3(1–40) gave rise to two products of lower mobility in an SDS/polyacrylamide gel, one corresponding to an HMGB1 dimer and the second (asterisk), to a product containing HMGB1 and H3(1–40) (Figure 5b and c, lane 3). The increased mobility of HMGB1 and some of its truncated products after treatment with EDC has been observed previously (see above) and is tail-length dependent. The HMGB1 dimer band is stronger in the presence of DNA [and independent of the presence of H3(1–40)], presumably because binding to DNA brings the HMGB1 molecules into closer proximity.

In the absence of DNA, cross-linking to H3(1–40) is significantly reduced on removal of the five C-terminal residues of HMGB1 (HMGB1Δ5) and was completely lost on removal of 10 residues (HMGB1Δ10) [Figure 5b(i) lanes 5 and 7, respectively], consistent with previous observations (17,18). In contrast, when DNA was present, these deletions did not significantly reduce the amount of cross-linked product [Figure 5c(i), lanes 5 and 7, respectively]. Some cross-linking was still detectable when only five tail residues remained (HMGB1Δ25), but not with the tailless protein [HMGB1Δ30; Figure 5c(i), compare lanes 11 and 13]. The HMGB1 dimer band was present for all tail-lengths but not for the tailless protein. The reduced cross-linking with the proteins containing shorter tails is presumably due to fewer carboxyl groups being available both for interaction with H3(1–40) and for reaction with EDC.

These results show that binding of the HMG boxes to DNA competes away the intramolecular interactions with the acidic tail, displacing the tail from the boxes. In the case of the truncated tails, in which most or all of the acidic residues are completely shielded by the boxes, displacement is necessary to allow them to interact with H3. In the full-length protein, although most of the tail is also tightly sequestered by the HMG boxes, cross-linking to H3(1–40) is nonetheless possible even in the absence of DNA because in this case the five or so C-terminal residues of HMGB1 are only loosely bound (19) and are thus available for interaction.

### H3(1–40) competes with intramolecular interactions in HMGB1 for binding to the acidic tail

**Figure 6.**
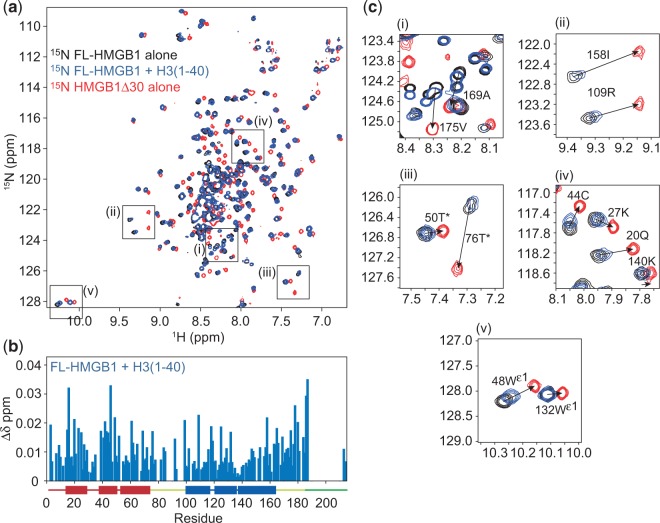
Interaction of H3(1–40) with HMGB1. (**a**) Overlay of ^15^N-HSQC spectra of HMGB1, HMGB1 on addition of H3(1–40) to a molar ratio or 1:1 and HMGB1 lacking the acidic tail and therefore representative of the fully ‘tail-free’ state (Δ30; red). (**b**) Chemical-shift perturbations (Δδ) are displayed for each residue that could be assigned; the noticeable gap spanning most of the acidic tail (187–212) was due to lack of sequence-specific assignments. (**c**) Representative examples of peaks that shift on addition of H3(1–40) to HMGB1 (compare black and blue). Peaks that shift do so along the same trajectory as those in the tailless protein (Δ30; red), indicated by arrows, suggesting that the addition of H3(1–40) reduces the fraction of the ‘tail-bound’ form. Peaks marked with an asterisk are aliased in the ^15^N dimension.

To investigate further, the interaction between the acidic tail of HMGB1 and the N-terminal tail of H3, NMR chemical-shift perturbation mapping experiments were carried out using ^15^N-labelled HMGB1 and unlabelled H3(1–40). The ^15^N-HSQC spectra of HMGB1 alone (black) after addition of H3(1–40) at a molar ratio of 1:1 (blue) are shown in Figure 6a. The spectra are generally similar, indicating that there is no significant structural change in HMGB1. However, many of the resonances throughout both the HMG boxes and the basic linkers shift by a small amount; the chemical-shift differences (Δδ) for each assigned residue are plotted in Figure 6b. (Resonances that can be attributed to the tail, although not assigned sequence-specifically, also shift to a small extent.) These changes can be attributed to binding of H3(1–40) to the acidic tail of HMGB1, competing with the intramolecular tail-box interactions, and causing consequent widespread chemical-shift changes as a knock-on effect. [A similar effect was observed on interaction of the linker histones H1 and H5 with the acidic tail in HMGB1 (11).] Consistent with this interpretation, overlaying the spectra with that of tailless HMGB1 (Δ30; Figure 6a, red) showed that binding of H3(1–40) caused peaks to shift towards the equivalent peaks in the tail-less protein; representative examples are shown in Figure 6c. The peaks shift only a small distance along this trajectory, presumably because H3(1–40) competes ineffectively with the intramolecular interaction.

**Figure 7.**
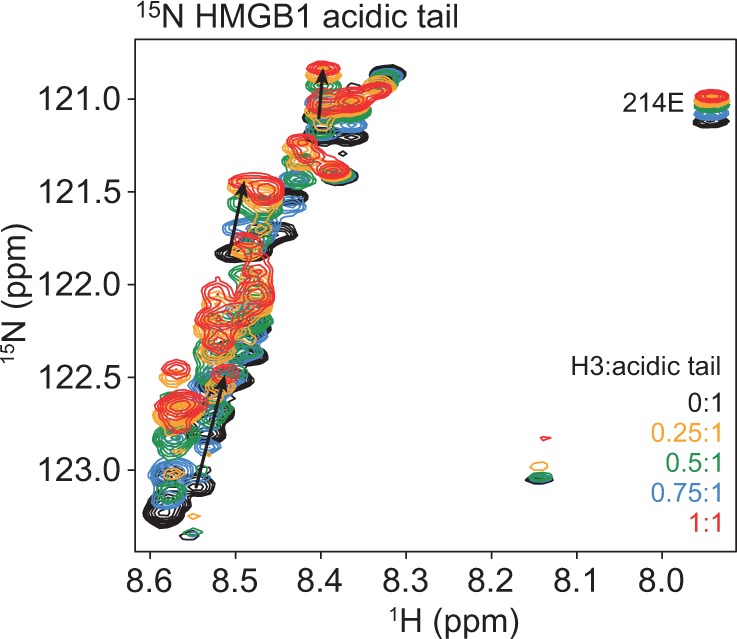
Interaction of H3(1–40) with the HMGB1 acidic tail peptide. ^15^N-HSQC spectra of the HMGB1 acidic tail peptide alone and on addition of H3(1–40) to the following H3:acidic tail molar ratios: 0.25:1, 0.5:1, 0.75:1 and 1:1. The progression of three representative peaks during the titration is indicated with arrows.

Since it was not possible to gain much direct information on H3(1–40) binding to the acidic tail in full-length HMGB1, due to spectral overlap and lack of tail resonance assignments, a similar titration was performed using a ^15^N-labelled acidic tail peptide (residues 185–214 of HMGB1). The ^15^N-HSQC spectrum of the HMGB1 tail (black) is shown in Figure 7. There is little dispersion of the resonances, indicating that the tail peptide is intrinsically disordered, as shown previously (19,39). Due to the higher flexibility of the tail as a free peptide, the peaks are now relatively sharp and resolved, but the low sequence complexity and lack of distinct sequential NOE information again preclude sequence-specific assignments. Upon addition of H3(1–40), most of the peaks shift, indicating that the interaction of H3(1–40) with the acidic tail is extensive and presumably competes with the equally extensive interactions of the acidic tail with the HMG boxes and basic linkers (19,20).

**Figure 8.**
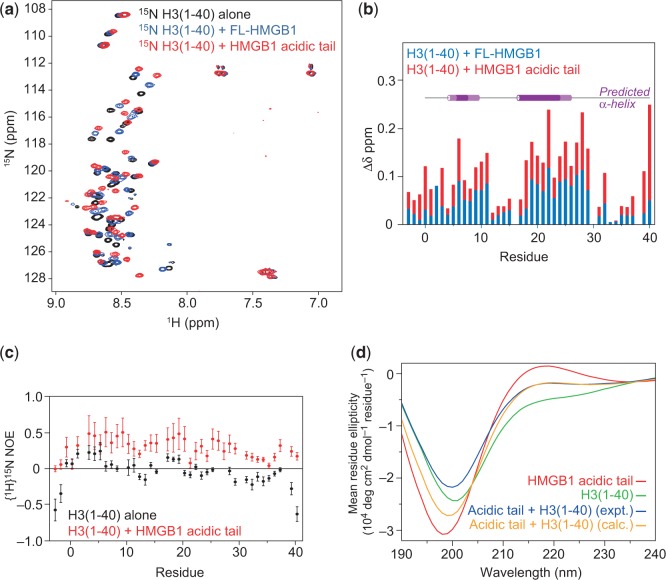
Interaction of HMGB1 and the acidic tail peptide with H3(1–40). (**a**) Overlay of the ^15^N-HSQC spectra of H3(1–40) alone and in the presence of HMGB1, or the acidic tail peptide at a 1:1 molar ratio. (**b**) Chemical-shift perturbations for all non-proline residues in H3(1–40) on interaction with HMGB1 or acidic tail peptide. Helical regions predicted by Jpred are shown above (consensus between algorithms in dark purple; maximum extent in light purple). (Residues <1 remain after thrombin cleavage of the His-tag and are not part of the H3 sequence.) (**c**) {^1^H}^15^N Heteronuclear NOE values for all non-proline residues in the H3(1–40) peptide alone (black circles) and on addition of acidic tail peptide (red circles). (**d**) Far-UV CD spectra of the individual acidic tail peptide, H3(1–40) and the 1:1 mixture of the two peptides. The sum of the spectra for the two individual peptides normalized for concentration was calculated to determine whether any structural changes occur on interaction.

To determine whether the tail in intact HMGB1 and the acidic tail peptide interact in the same way with H3(1–40), unlabelled HMGB1 and acidic tail peptide were titrated into ^15^N-labelled H3(1–40) and ^15^N-HSQC spectra were recorded (Figure 8a). The H3 N-terminal tail (black) is unstructured, as indicated by the narrow chemical-shift dispersion and low heteronuclear NOE values [see below; Figure 8c)]. On titration with full-length HMGB1 or the acidic tail peptide, many resonances show significant progressive shifts, indicative of an extensive interaction in fast exchange on the chemical-shift timescale. Interestingly, both sets of shifts lie on the same trajectory, suggesting that the mode of interaction in each case is similar. The shifts caused by the acidic tail peptide are greater, suggesting that the free tail binds to H3(1–40) with higher affinity than the tail in HMGB1, not surprisingly since this is sequestered by the HMG boxes and basic linkers (19,20). Chemical-shift differences (Δδ) for each assigned residue on addition of either HMGB1 or acidic tail peptide (1:1) are overlaid in Figure 8b. Intriguingly, the most significant shifts cluster into two patches roughly spanning residues 2–11 and 19–29, which contain several residues that are known to be post-translationally modified (40), but no significant shifts were observed around residues K36 and K37, which have been reported as the site of EDC cross-linking with the HMGB1 tail (18).

The interacting patches overlap with the helical regions predicted by Jpred (Figure 8b) (41). Further, {^1^H}^15^N heteronuclear NOE measurements of the H3 tail in the absence and presence of the HMGB1 acidic tail show that on interaction H3(1–40) becomes conformationally more constrained (Figure 8c, compare black and red circles). In the absence of the HMGB1 tail, H3 displays {^1^H}^15^N NOE values around zero, indicative of significant motions on a ns-ps timescale and characteristic of a disordered peptide; on addition of the HMGB1 tail, the values increase significantly to 0.2–0.5, approaching the values seen in stable secondary structures (>0.6). Interestingly, higher {^1^H}^15^N NOE values are observed across the entire peptide, and not just the sites of interaction determined by chemical-shift perturbation mapping or the predicted helical regions (Figure 8b), showing that binding is accompanied by a global loss in flexibility.

To establish whether the H3/acidic tail interaction is accompanied by specific structural changes, CD spectra of the H3 and HMGB1 tail peptides, separately and together, were compared (Figure 8d). (Tail peptides were used so that any small changes would not be obscured by large helical signals present in the full-length proteins.) The spectra of the isolated peptides indicate that they are intrinsically disordered. The acidic tail of HMGB1 gives a classic random-coil spectrum with a large negative ellipticity in the far-UV and a small positive ellipticity around 218 nm. The H3 spectrum also displays a random-coil minimum in the far-UV, albeit at a slightly higher wavelength, but there is also a small negative ellipticity at 222 nm, suggesting the presence of a low amount of α-helix. When the spectrum of the peptide mixture is compared with the theoretical spectrum calculated from the separate components, the only differences observed are around the random coil minimum, indicating that the two peptides interact, and that the interaction alters the conformational sampling, but this is not accompanied by any significant gain of identifiable secondary structure.

## DISCUSSION

Here we confirm that HMGB1 interacts primarily with H3 in chromatin and that the acidic tail of HMGB1 and the basic tail of H3 are key players. Further, we characterize in detail the interaction between these two intrinsically disordered regions.

Sucrose-gradient sedimentation, biotin label-transfer and EDC cross-linking show that HMGB1 binds in proximity to, and interacts with, H3 in chromatin and that the acidic tail of HMGB1 and the N-terminal tail of H3 are involved (Figures 2, 3b and 4). Moreover, use of a series of HMGB1 tail-truncated derivatives shows that an acidic tail of only five residues can still be cross-linked to H3 when HMGB1 is bound to chromatin (Figure 4). This is in contrast to previous studies where EDC cross-linking of free HMGB1 and a similar set of truncated proteins with purified core histones suggested that the C-terminal 5–10 residues of the HMGB1 tail were critical for interaction with H3 (17,18). These differences can be reconciled by the observation that although EDC cross-linking of H3(1-40) with HMGB1 and its truncations in the absence of DNA does require the C-terminal 5–10 acidic tail residues, in the presence of DNA all the proteins that possess any acidic tail, however short, can be cross-linked to H3(1–40) (Figure 5). We conclude that binding of the HMG boxes to DNA frees the acidic tail from intramolecular interactions with the DNA-binding surfaces (19,20,38), allowing the shorter acidic tails to interact with H3.

Detailed mapping of the interaction using NMR chemical-shift perturbation shows that H3(1–40) can compete with the intramolecular box-tail interactions in HMGB1, but binds much more tightly to the free HMGB1 acidic tail peptide (Figures 6 and 7). Most of the residues in the acidic tail are involved, confirming that the interaction is more extensive than previously thought. The {^1^H}^15^N NOE and chemical cross-linking taken together suggest a dynamic 1:1 complex. We have previously shown a similar competitive (intermolecular) interaction between the acidic tail of HMGB1 and the basic C-terminal tails of the linker histones H1/H5 (11).

Interestingly, the interaction of the acidic tail with H3(1–40) involves two regions of the H3 tail (residues 2–11 and 19–29) that are predicted to be helical. However, CD spectroscopy and heteronuclear NOE measurements (Figure 8) together show that although the intrinsically disordered peptides become more conformationally constrained when they interact, presumably through a predominantly electrostatic interaction, they do not appear to adopt any stable secondary structure, at least as judged by lack of significant chemical-shift dispersion in a ^15^N-HSQC spectrum and lack of change in the 208–222 nm region of a CD spectrum. (It is of course possible that in chromatin, the presence of DNA induces a disorder-to-order transition in H3.) Examples where both partners remain unstructured on interaction (42) are rare, as noted (43), in contrast to the many cases in which a disordered protein remains unstructured on interaction with a folded partner (e.g. 44–46). There are more documented examples of the ‘classical’ situation in which an unstructured protein undergoes a disorder-to-order transition on interaction with a structured partner-protein or DNA [reviewed by Dyson and Wright (47)].

**Figure 9.**
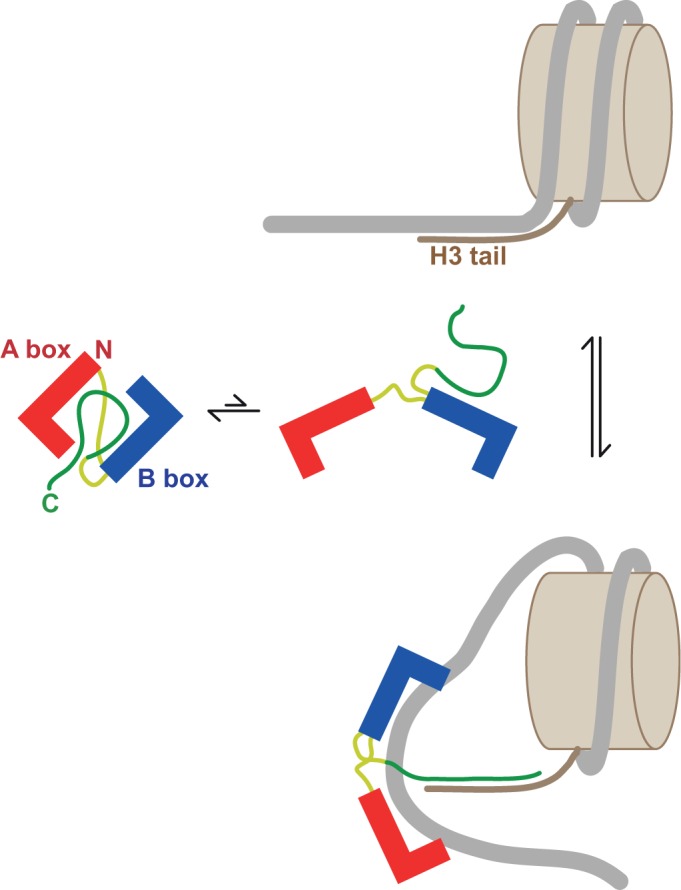
Proposed mechanism for the interaction of HMGB1 and H3 in chromatin. HMGB1 is shown in its closed (auto-inhibited) and open (binding-competent) forms (see Figure 1). HMGB1 binds to the linker DNA at the DNA entry/exit point of the nucleosome, close to where the H3 N-terminal tail exits between the two gyres of DNA, and binding is stabilized by acidic tail/H3 tail interactions. A combination of (i) disruption of H3 tail/DNA interactions, and (ii) HMG-induced untwisting of DNA to produce a positive writhe that promotes unwrapping of DNA, result in destabilization of the nucleosome core.

In chromatin, HMGB1 binds to the linker DNA at the DNA entry/exit point of the nucleosome, close to where the H3 N-terminal tail exits from between the two gyres of DNA (48). Binding of HMGB1 to chromatin is likely to compete with H3 tail/linker DNA interactions and concomitantly release the HMGB1 acidic tail from intramolecular interactions with the HMG boxes and linkers; the two domains would then be free to interact. The H3 tail plays an important role in stabilizing nucleosome structure; removal of the tail leads to increased nucleosome sliding and accessibility of DNA at the edges of the nucleosome, and destabilizes the interaction with the H2A/H2B dimer (49). In addition, mutation of the acetylatable lysine residues in the H3 tail to glutamine to mimic acetylation, which would be expected to weaken H3-tail DNA interaction, also increased nucleosomal DNA accessibility (50). The combination of competition for H3 tail/DNA interactions and the bending and untwisting of DNA that occurs when the HMG boxes bind may therefore act to destabilize DNA-octamer contacts and potentially stabilize bent DNA structures (Figure 9); these might provide substrates for remodelling complexes [such as ACF/CHRAC (14)]. The related single HMG-box protein, HMG-D, indeed enhances accessibility of nucleosomal DNA at one side of the nucleosome and at a site near the dyad, as judged by endonuclease sensitivity (51), and removal of the (short) acidic tail abolishes this effect. Interestingly, the interaction of the tailless, single-box, yeast protein Nhp6a with chromatin alters DNA accessibility at sites in the nucleosome core but not at the DNA entry/exit points (52). It is likely that interaction of the acidic tail of HMG-box proteins with the basic N-terminal tail of H3 is important for positioning the protein such that the DNA at the entry/exit point is additionally destabilized.

The interaction of HMGB1 with chromatin might be fine-tuned by post-translational modifications (5,6). The H3 tail is known to be subject to a plethora of different post-translational modifications (40), which, in addition to recruiting specific proteins, might affect binding to linker DNA and HMGB1. HMGB1 is also known to be acetylated (53,54) and phosphorylated (55). These modifications might modulate the intramolecular interactions as well as directly affecting binding of partners.

In summary, we have demonstrated that the acidic tail of HMGB1 is released on binding of the HMG boxes to chromatin, and that its subsequent interaction with the N-terminal tail of H3 is extensive, resulting in a complex that lacks detectable order. This currently poorly defined, yet specific, interaction serves to localize HMGB1 binding near the nucleosome dyad, where the induced bending and untwisting of DNA is likely to destabilize nucleosomes, increasing DNA accessibility.

## FUNDING

Biotechnology and Biological Sciences Research Council [BB/D002257/1 to J.O.T.]; Research Studentship (to L.C.). Funding for open access charge: from laboratory funds.

*Conflict of interest statement*. None declared.
